# Electroactivity of polyphenols in sweet sorghum (*Sorghum bicolor* (L.) Moench) cultivars

**DOI:** 10.1371/journal.pone.0234509

**Published:** 2020-07-14

**Authors:** Minori Uchimiya, Joseph E. Knoll

**Affiliations:** 1 USDA-ARS Southern Regional Research Center, New Orleans, Louisiana, United States of America; 2 USDA-ARS Crop Genetics and Breeding Research Unit, Tifton, Georgia, United States of America; Institute for Biological Research "S. Stanković", University of Belgrade, SERBIA

## Abstract

Polyphenols and other potential health-promoting components of sorghum (*Sorghum bicolor* (L.) Moench) drove its recent growth in the U.S. consumer food industry. Linear sweep (cyclic voltammetry, CV) and differential (cyclic differential pulse) voltammetry methods were developed to detect target polyphenols and amino acids in sweet sorghum juice without interference from the dominant secondary (trans-aconitic acid) and primary (sucrose) metabolites. Of 24 cultivars investigated, No.5 Gambela showed the highest electron-donating capacity, as indicated by the highest peak area, height, and peak anodic potential. Pearson’s correlation analysis indicated the contribution of polyphenols (rather than amino acids) on CV voltammograms of juice samples. The E_h_-pH values of 173 sweet sorghum juice samples collected in 2017 aligned with quercetin model polyphenol. Accumulation of quercetin-like polyphenols in No.5 Gambela could offer antioxidant-rich juice for conversion to edible syrup as well as an increased tolerance against a recently emerged pest, sugarcane aphid [(*Melanaphis sacchari* (Zehntner)].

## Introduction

Over the past several years, sorghum (*Sorghum bicolor* (L.) Moench) production for the consumer food industry has drastically grown within the U.S [1]. This market trend originates from high phenolics in sorghum grain, along with other attributes including gluten-free and high-fiber characteristics. A wide range of higher plants produce polyphenols as the secondary metabolites for the defense against photooxidative damage and pests [2]. Sorghum genotypes could be bred to accumulate desirable phenolics for a wide range of food products including sweeteners (for sweet sorghum varieties), cereals, and antioxidant food additives. However, the current bottleneck in precision breeding is the access to field-deployable omics fingerprinting tools to phenotype crop varieties without the analyte separation required for metabolomics. Such chemistry tool must be simple, rapid, and user-friendly to the breeders.

Redox chemistry underlies the antioxidant properties of polyphenols [2]. Thermodynamic driving force (standard reduction potential, E^0^ in volts) and kinetic lability determine the redox reactivity of polyphenols [3, 4]. Aromatic rings of polyphenols (1) decrease E^0^ by both inductive and resonance effects, (2) stabilize the radical intermediates by resonance stabilization, enabling reversible electron transfer, and (3) protect the phenolic redox center from nucleophilic addition and other side reaction-induced decomposition [4]. Polyphenols (those containing a quinone redox center) and flavins are well-described electron shuttles in biological systems, because of their ability to transfer one or two electrons reversibly between the bulk electron donor and the terminal electron acceptor [5]. Sorghum and other polyphenol-rich cereals, fruits, and vegetables contain a complex mixture of phenolic and non-phenolic electroactive structures [2]. In addition, the polyphenol composition is highly cultivar-dependent [6].

Our previous report used cyclic voltammetry (CV) to oxidize the stem juice of sweet sorghum; polyphenolic structure similar to quercetin was determined to be the primary redox-active moiety [7]. A sweet sorghum genotype called No.5 Gambela showed resistance to a recently emerged pest in the U.S.: sugarcane aphid [(*Melanaphis sacchari* (Zehntner)]. No.5 Gambela accumulated polyphenols as well as a carboxylate secondary metabolite, trans-aconitic acid [8]. Trans-aconitic acid was highly sensitive to spectrophotochemical detection by fluorescence excitation-emission (EEM) and UV/visible absorption, within the wavelength ranges overlapping with polyphenols [8]. Trans-aconitic acid and polyphenols [9] in juice, riboflavin-like fluorophore in bagasse [8], and dhurrin (tyrosine-derived cyanogenic glycoside) in leaves [10] are all candidate allelochemicals against aphids. Electrochemistry-based fingerprinting methods are necessary to understand the importance of redox-active defense phytochemicals.

Square wave (SWV) [11–13] and differential pulse voltammetry (DPV) [14], and CV [15] have been used to detect aliphatic carboxylates including trans-aconitic, fumaric, maleic, malic, oxalic, and citric acids. The SWV of fumaric and maleic acids [11, 12] were irreversible in neutral and acid media, and the voltammograms resembled that of trans-aconitic acid [13], which was most electroactive in the fully protonated form. Differential electrochemical methods (DPV and SWV) are used to improve the detection limit of linear sweep voltammetry (CV) [16] for the target electroactive structures in food and beverage [17]. Differential electrochemical methods improve the signal to noise ratio by minimizing the capacitive current [16]. This is achieved by (1) sampling current after capacitive current has decayed, or (2) taking the differential currents [16]. Faradaic process transfers charge across the interface to give a steady-state current with distinct peaks [18]. In contrast, capacitive current originates from the charging and discharging of the electrode double layer to give a box-shaped voltammogram [19]. Capacitive current is also called the background current (without redox-active species), double layer current, or non-Faradaic current to describe the rearrangement of ions in the double layer when potential is applied to the working electrode [19]. Magnitude of background current is directly proportional to the scan rate, while the current from diffusion-controlled electrochemical reaction is proportional to the square root of the scan rate [20]. As a result, background (capacitive) current is the major source of noise in CV especially at fast scan rates [16]. In addition, double layer capacitance is controlled by the electrode potential, which depends on the electrode material and the type and concertation of the supporting electrolyte [16].

The objective of this study was to understand the redox reactivity of secondary metabolites putatively contributing to the pest resistance of sweet sorghum cultivar, No.5 Gambela [8]. No.5 Gambela accumulated trans-aconitic acid and polyphenols, but not amino acids [8]. Because those classes of chemicals have overlapping peaks in fluorescence and UV/visible detection [8], electrochemistry was investigated in the present study as a tool to differentiate polyphenols from amino acids and trans-aconitic acid. Such redox method would allow breeding of sorghum cultivars utilizing different mechanisms: antioxidants vs. organic acids. First, DPV was employed to increase the sensitivity of detection for the peak previously attributed to quercetin-like structures in sweet sorghum juice. Peak identification relied on the comparison of juice against electroactive structures in juice or bagasse of sweet sorghum [7, 8]: tryptophan, tyrosine, trans-aconitic acid, quercetin, and riboflavin.

## Materials and methods

Sweet sorghum cultivars were planted in April, May, or June 2017, and harvested at the hard dough stage of grain fill, which occurred during July through November, depending on cultivar and planting date. Field experimental design and juice characterization procedures were described in detail previously [7, 21, 22], and are summarized in Section I of S1 File. Group membership and Plant Introduction (PI) number [23] of 24 sweet sorghum genotypes examined in this study are provided in S1 Table in S1 File.

### Juice and bagasse characterization

As described in detail previously [7, 21], distilled, deionized water (DDW) with a resistivity of 18 MΩ cm (APS Water Services, Van Nuys, CA) was used in all laboratory procedures. All chemical reagents were obtained from Sigma-Aldrich (Milwaukee, WI) with the highest purity available. Because sweet sorghum juice is unstable at room temperature [24], samples were defrosted in a refrigerator, and were immediately diluted (large-orifice pipette tips, Fisher, Hampton, NH) by DDW, filtered (0.45 μm PVDF, Fisher), and analyzed. Brix (estimate of g sucrose/100g solution) was measured using a portable digital refractometer (300053; Sper Scientific, Scottsdale, AZ). Sucrose, glucose, fructose, and citric, oxalic, trans- and cis-aconitic acid concentrations were quantified using an HPLC system with refractive index and diode array detectors (Agilent Technologies, Santa Clara, CA) and a Hi-Plex H column (Fisher) with 5 mM sulfuric acid mobile phase at 0.6 mL min^-1^ flow rate and 20 μL injection volume. Total organic carbon (TOC in gC/L) and total nitrogen (TN in gN/L) were analyzed using a Torch combustion TOC/TN analyzer (Teledyne Tekmar, Mason, OH). UV/visible spectra (HP8452A, Hewlett-Packard, Palo Alto, CA) of juice (20-fold diluted and 0.45 μm filtered) were obtained with DDW blank subtraction.

Electric conductivity (EC), pH, and oxidation reduction potential (ORP; E_h_ in mV) of juice samples were determined without dilution using YSI 3200 conductivity meter (YSI, Yellow Springs, OH) for EC; ORION ROSS sure-flow glass combination pH electrode for pH; and an epoxy sure-flow combination redox/ORP electrode with built-in temperature probe (9678BNWP, Fisher) for E_h_. Sartorius Professional PP-15 meter (Sartorius, Bohemia, NY) was used to record the electrode response for both pH and E_h_. The ORP electrode is composed of platinum sensing electrode and Ag/AgCl reference electrode. At 25 °C and 4 M KCl filling solution employed, E_h_ value relative to the standard hydrogen electrode (SHE) is calculated by adding 200 mV (±60 mV, error originating from variations in sample ionic strength and reference junction potentials) to the absolute value measured using ORP electrode.

Fluorescence EEM spectra of juice and bagasse samples were collected using F-7000 spectrofluorometer (Hitachi, San Jose, CA) at 220–500 nm excitation and 280–730 nm emission wavelengths in 3 nm intervals; 2.5 nm excitation and emission slits; auto response time; and 2400 nm min^-1^ scan rate [8]. After blank subtraction and correction for Rayleigh and Raman peaks, parallel factor (PARAFAC) analysis was performed with non-negativity constraint using MATLAB version 8.6.0.267246 (R2015b; Mathworks, Natick, MA) with PLS toolbox version 8.6.2 (Eigenvector Research, Manson, WA). PARAFAC models contributions of primary fingerprints on each sample by minimizing the sum of squares of the residuals. For 2017 juice samples, three PARAFAC components (based on residual/leverage analysis) were assigned to tryptophan-like (hereby denoted EEM/PARAFAC factor 1), tyrosine-like (factor 2), and phenolic structure with high aromaticity (aromatic factor 3) [8]. EEM/PARAFAC results were interpreted as follows: a juice sample (representing a cultivar and a planting month) with high absolute contribution from factor 1 is estimated to have high “concentration” of tryptophan-like structure. Standard curve calibration of colorimetric method is not used in EEM/PARAFAC, because of a complex mixture of contributing structures, e.g., different phenolic molecules. The purpose of fluorescence technique is to estimate the relative fraction of target analyte in a large set of samples, without analytical separation required for LC-MS used in metabolomics.

### Electrochemistry

The CV and CDPV of diluted (2-fold by DDW) juice samples (buffered at pH 5 with 40 mM phosphate in 0.1 M KCl) were obtained using a WaveNow potentiostat with disposable screen-printed carbon electrodes (SPE with Ag/AgCl reference electrode; Pine Research Instrumentation, Durham, NC) of two different surface areas: 2 mm diameter disk, and 4×5 mm rectangle patterned carbon working electrode. The larger surface area of the 4x5 mm carbon SPE is expected to enhance the current signal relative to the 2 mm SPE used in our previous report [7]; however, the background current could increase as well. Both SPEs are compatible with aqueous solution, and are composed of polyethylene terephthalate plastic screen with Ag/AgCl reference electrode and carbon working/counter electrodes on conductive silver layer. For each juice sample, a new SPE was used to first collect background CV (0.1 M KCl and 40 mM phosphate buffer at pH 5) by applying -0.5 V for 60 s, and subsequently increasing the potential to 1.2 V, and then decreasing to -0.5 V at 100 mV s^-1^ sweep rate in both directions. Immediately after collecting the background voltammogram, CV of the juice sample was obtained using the same SPE.

To identify electroactive structures in juice samples, 1 mM of following standards were prepared in DDW to perform CV as described above: tryptophan, tyrosine, trans-aconitic acid, sucrose, and citric acid. CV of quercetin (as a model polyphenol) was obtained from the literature employing 0.5 mM quercetin dissolved in 50 vol% ethanol [7]. Quercetin is sparingly soluble in water and reported aqueous solubility varies by orders of magnitude [25]. Carbon SPE employed in this study is designed for aqueous solutions, and compatibility with organic solvents decreases in the following order: ethanol>acetonitrile>methylene chloride. The layer containing working/reference/counter electrodes begins to swell after a few minutes in methylene chloride, but not in acetonitrile. Blank subtraction was utilized to account for co-solvent and other background effects, as described in detail below.

CDPV employed a procedure analogous to CV with the following pulse parameters: 50 mV height, 10 ms width, 100 ms period, 10 mV increment, and 3 ms pre- and post-pulse widths. This parameter setting corresponds to the effective scan rate (increment*(1/period)) of 100 mV/s. Higher mV height (90 instead of 50) or wider mV (upto 1500 mV) did not improve the peak sensitivity or resolution. For CV as well as CDPV, current (in mA) range was first determined using the auto-range option of the electrochemical software (AfterMath version 1.5.9568, Pine Research Instrumentation) to auto-adjust current and voltage. This procedure prevents low sensitivity leading to the lack of peak, or excessive sensitivity resulting in the clipped/truncated peak. Once the amplitude range was determined, the range was manually set to 10 μA for both CDPV and CV to prevent current drifts caused by switching cell/circuit during auto-ranging. In addition, 60 s induction period (at -500 mV) was used to allow the cell to equilibrate with the initial signal level applied to the working electrode. Above 1 V, solvent (water) oxidation and reactions involving O_2_ will influence the anodic current [26].

Each raw anodic CV and anodic/cathodic CDPV (in current (A) vs. potential (V)) was processed using OriginPro 2019 (OriginLab, Northampton, MA) by (1) background subtraction to minimize background signals from conductive carbon ink and other electrochemical cell components, (2) Savitzky-Golay smoothing of the first derivative to enhance peak resolution, and (3) trapezoidal integration at 0.5–1.0 V range. The derivative CV and CDPV of juice sample was additionally processed by the Gaussian integration (OriginPro 2019) to obtain the peak anodic/cathodic potential (E_pa_ and E_pc_ as the center of peak in volts) and corresponding peak area and maximum current height in mA. Gaussian fitting of peaks employed user-selected baseline anchor points connected by interpolation; peak selection by the second derivative; Gaussian fit until convergence; and error analyses by the reduced Chi-square and residual plots.

### Statistical analysis

Main effects and interactions were examined by factorial (cultivar×planting month) ANOVA using Statistica version 12 (Statsoft, Tulsa, OK) at a significance level of p<0.05. Type VI sums of squares was used to test the effective hypothesis for unbalanced observations. If significant differences existed, post hoc comparison by Tukey’s honestly significant difference (HSD) test was performed with cultivar or planting month as the categorical factor.

To examine linear relationships between all investigated parameters for the 2017 juice and bagasse samples, a Pearson’s correlation matrix pseudocolor map was constructed using MATLAB version 8.6.0.267246 (R2015b; Mathworks, Natick, MA) with PLS toolbox version 8.7 (Eigenvector Research, Manson, WA). Variables were reordered by the similarity in Pearson’s r values using a modified k-nearest neighbor algorithm.

Eq 1 [27] was used to explore the linear relationships between the anodic current (1^st^ derivative) of juice samples against the following parameters that previously [8, 28] showed correlation with electrochemical parameters, and could directly or indirectly contribute to redox reactivity of juice samples (x_i_): absolute contributions of aromatic, tryptophan, and tyrosine EEM/PARAFAC fingerprints; sucrose, trans-aconitic acid, and citrate concentrations; UV/visible absorbance at 270 and 340 nm; and Brix, pH, EC, E_h_, TN (in gN/L).

$$r=\frac{n\left(\sum x_{i}y_{i}\right)-\left(\sum x_{i}\right)\left(\sum y_{i}\right)}{\sqrt{\left[n\left(\sum x_{{}_{i}}^{2}\right)-{\left(\sum x_{i}\right)}^{2}\right]\left[n\left(\sum y_{{}_{i}}^{2}\right)-{\left(\sum y_{i}\right)}^{2}\right]}}$$ (1)

where x_i_ is the juice property, y_i_ is the current in mA (first derivative) at a given potential (in V), and n is 24 for juice samples representing each cultivar listed in Section II of S1 File.

## Results and discussion

### Electroactive components: Amino acids vs. polyphenols

**Fig 1:**
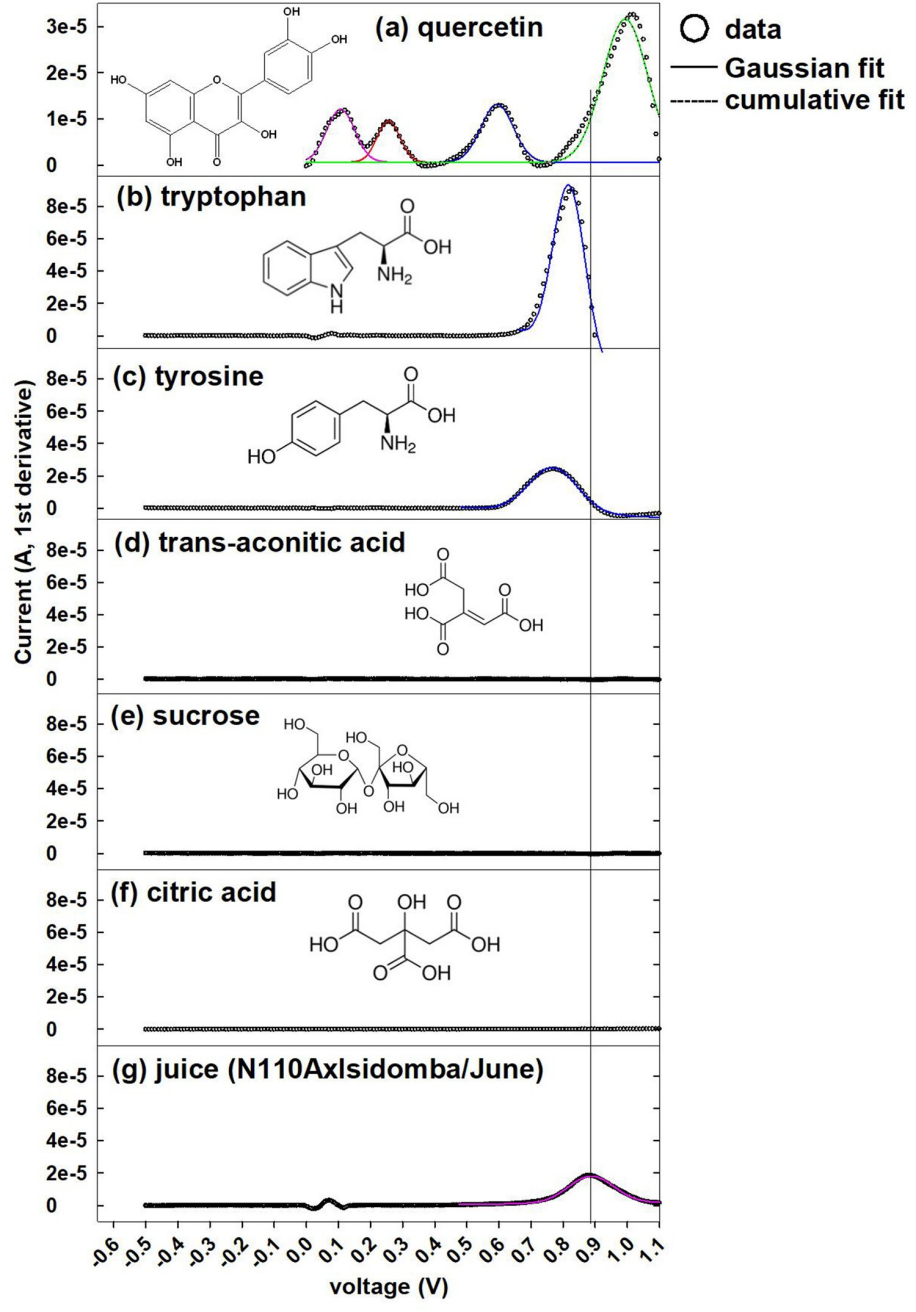
Raw CV (circles; after background subtraction and first derivative with smoothing) and Gaussian fits (colored lines) of 1 mM standards in DDW (a-f, quercetin (literature value) is 0.5 M in ethanol) [7] and representative juice sample (g) using 2 mm carbon SPE. Horizontal line represents E_pa_ of juice (0.81V in Table 2).

Fig 1g shows the anodic peak response recorded by CV for sweet sorghum juice sample. For comparison, analogous voltammograms for primary chemical structures in sweet sorghum juice are provided in Fig 1a–1f, where y-axis scale is fixed (except sparingly water-soluble quercetin in Fig 1a) to allow a visual comparison of peak heights. Trans-aconitic acid, sucrose, and citric acid did not show anodic peaks (Fig 1d–1f) within the potential range where sweet sorghum juice had a peak (Fig 1g). In contrast, both tryptophan and tyrosine showed a single peak within the voltage range overlapping with juice sample. In addition, a peak attributable to–OH group on the A-ring of quercetin occurred within the potential range of juice sample (Fig 1a and 1g). Quercetin contains five oxidizable–OH substituents. Experimentally observable peaks will depend on pH and other electrochemical parameters as well as data processing methods. Hendrickson et al. (1994) attributed three observable anodic peaks of quercetin to hydroxyl groups on the catechol B-ring, C-ring, and A-ring, towards more positive potentials [29]. Brett and Ghica (2003) observed a fourth peak originating from the second–OH on the catechol B-ring [30]. In the literature, total number of CV peaks for a given compound (quercetin in this case) is determined by visual inspection of a voltammetric curve lacking clear baseline. In Fig 1a, overlapping peaks at the least positive potential are attributable to two hydroxyl substituents on catechol B-ring of quercetin. Additional peaks towards more positive potentials are attributable to hydroxyls on C-ring and A-ring of quercetin. There are several complexities in assigning a polyphenol structure to anodic peak of juice in Fig 1g. First, sweet sorghum juice is expected to contain a complex mixture of polyphenols, rather than a single chemical structure such as quercetin. Second, hydroxyl substituents on a given phenolic structure give rise to multiple peaks at different potentials, as observed for quercetin in Fig 1a. Because of those complexities, bulk electron donating capacity of each juice sample will be quantified as the peak area at a given potential to compare different sorghum cultivars.

Fig 1 indicates that E_pa_ (potential at the center of anodic peak) could be used to differentiate polyphenols from amino acids in juice samples. S3 Table in S1 File provides E_pa_ and Gaussian-integrated peak area for tryptophan, tyrosine (1 mM in DDW), quercetin, and catechin (in 50 vol% ethanol) authentic standards [7]. The CV-derived E_pa_ values were lower for the amino acids (0.77–0.83 V) than juice (0.88±0.03 V as mean±s.d. for n = 170), and tryptophan had higher peak area than tyrosine. Analogous trends were observed for CDPV-derived E_pa_ values that are consistently lower than the CV. Model polyphenols catechin and quercetin had E_pa_ above and below that of juice.

To understand the relative contribution of polyphenols and amino acids in juice, S1 Fig in S1 File shows the anodic CV (a) and CDPV (c) of a cultivar containing high tryptophan- and tyrosine-like structures (N109AxChinese/April), based on the absolute contributions determined by fluorescence EEM/PARAFAC [8] described in Materials and Methods. This protein-rich cultivar was compared against another cultivar having high polyphenol-like structures (No.5 Gambela/June) in S1b and S1d Fig in S1 File. For CV (left panels in S1 Fig in S1 File), higher E_pa_ and peak area were observed for No.5 Gambela/June (0.93 V E_pa_ and 4.2e^-6^ peak area) than N109AxChinese/April (0.85 V E_pa_ and 1.4e^-6^ peak area). Analogous trends were observed for CDPV (right panels in S1 Fig in S1 File) comparing the same two cultivars. In conclusion, S1 Fig in S1 File indicates higher E_pa_ and peak area of a juice sample accumulating polyphenols (No.5 Gambela/June) than amino acids (N109AxChinese/April).

In addition to comparing individual juice samples in S1 Fig in S1 File, replicate samples of a given cultivar (across planting months) were used to observe the contribution of polyphenols, as opposed to amino acids. Cultivars having highest polyphenol-like structures (No.5 Gambela) had the highest E_pa_ by CV (anodic 0.5–1 V) of 0.93±0.01 V (mean±standard error, n = 7), as opposed to 0.87±0.01 V (n = 8) for N109AxChinese containing the highest amino acids [8]. For CDPV anodic current having wider E_pa_ range originating from Faradaic current, No.5 Gambela (0.84±0.01 V, n = 7) and Isidomba (0.82±0.01 V, n = 9) were significantly higher than N109AxN98 (0.76±0.01 V, n = 9). In conclusion, a cultivar enriched with polyphenols (No.5 Gambela) [8] had CV peak with higher E_pa_ attributable to polyphenol oxidation, while an amino acid-enriched cultivar (N109A×Chinese) had significantly lower E_pa_ that could originate from tryptophan and tyrosine. Subsequent section will further examine the genotype effects on relative importance of amino acid and polyphenol electroactive structures.

**Fig 2:**
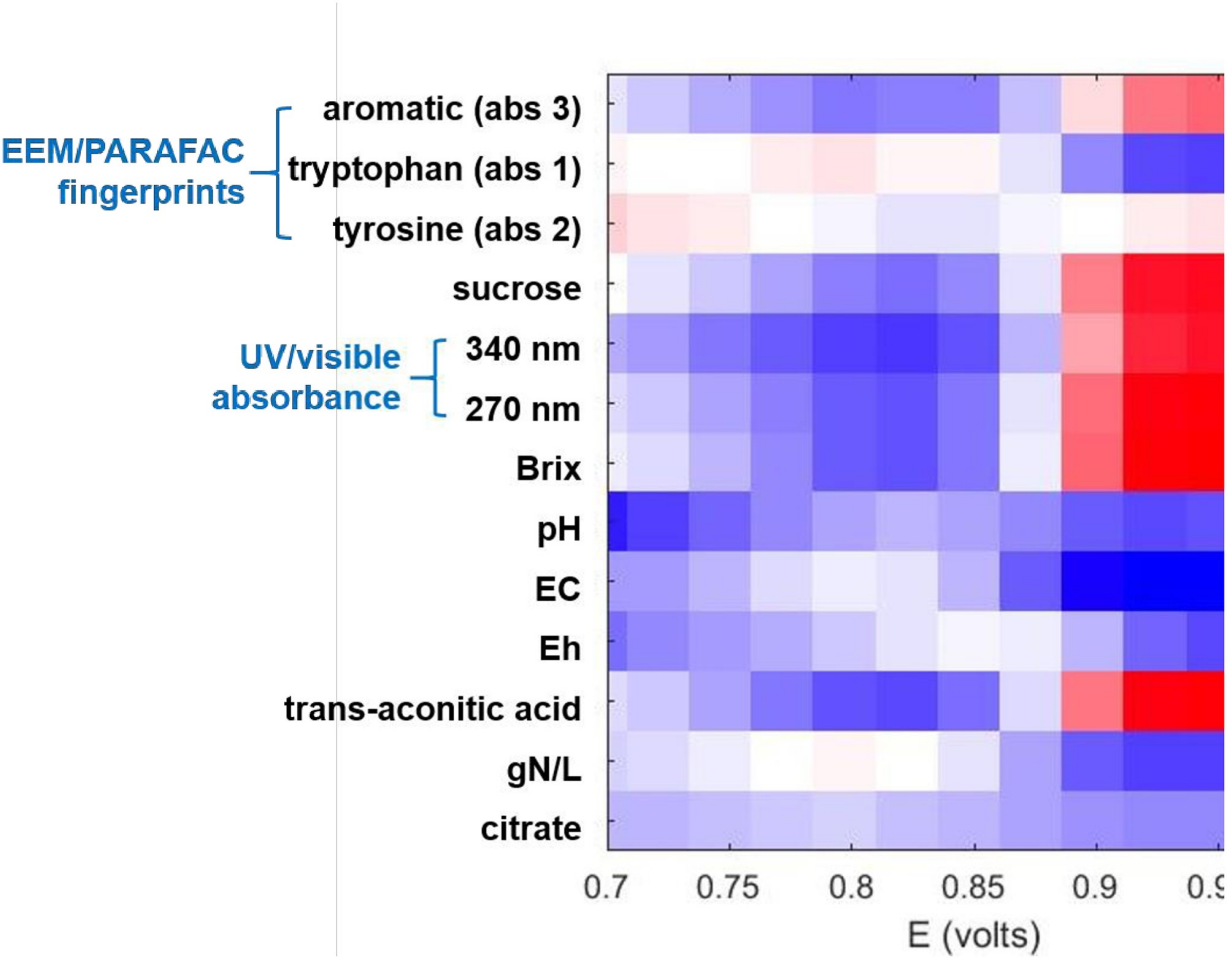
Pearson’s correlation coefficient between CV peak current and juice properties. Only juice peak voltage range was used.

**Fig 3:**
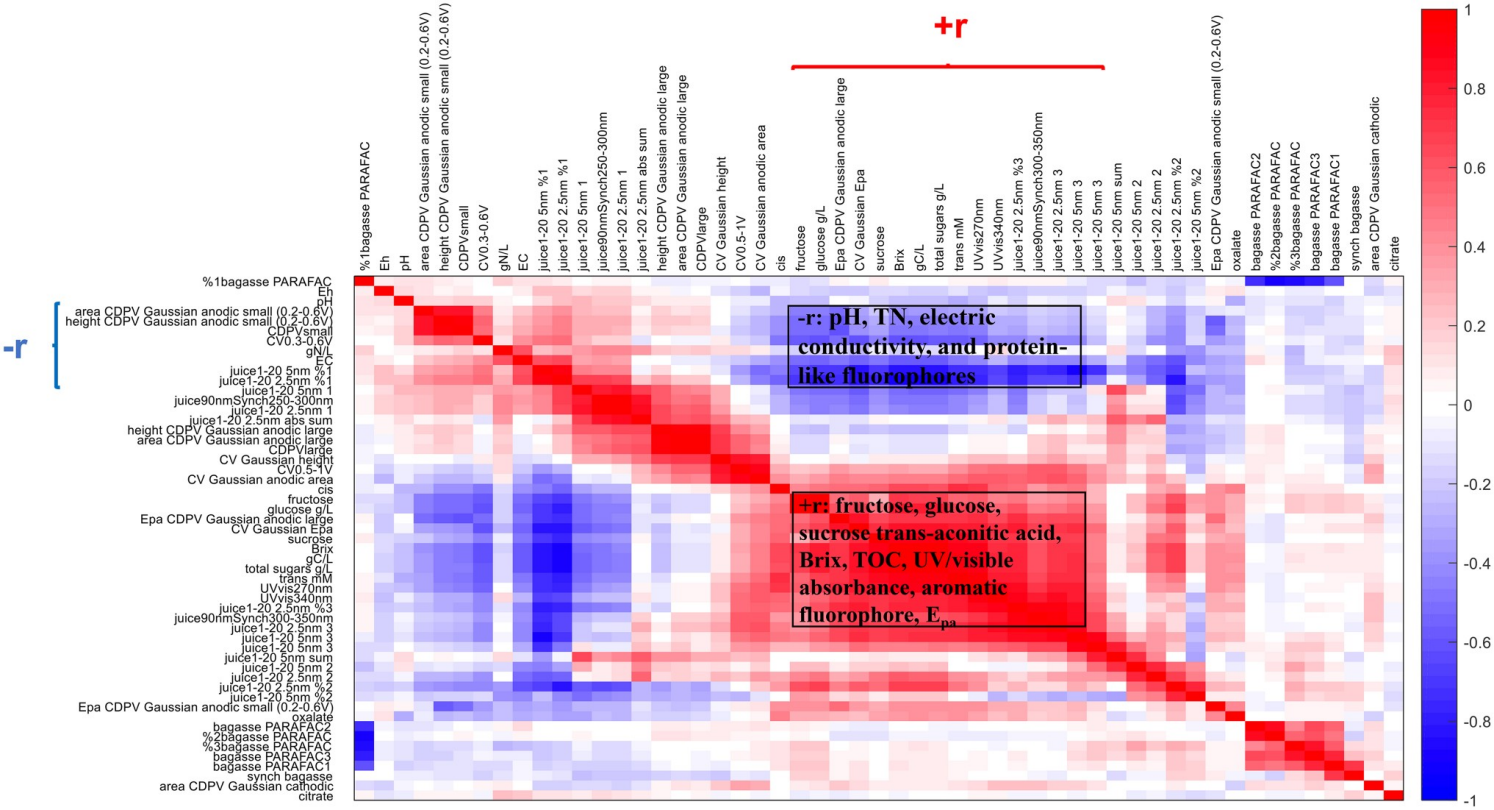
Pearson’s r values between all chemical analyses conducted on sweet sorghum juice and bagasse samples collected in 2017. Red regions indicate positive correlation (+r), and darker red indicates higher linearity; blue regions indicate negative correlation (-r). Parameters in +r region: fructose, glucose, sucrose, trans-aconitic acid, Brix, TOC, UV/visible absorbance, aromatic fluorophore, and E_pa_. Parameters in–r region: pH, TN, electric conductivity, and protein-like fluorophores.

Fig 2 shows Pearson’s correlation (Eq 1) between anodic current of juice and the following parameters: EEM/PARAFAC absolute contributions for aromatic, tryptophan, and tyrosine fingerprints; concentrations for sucrose, trans-aconitic acid, and citrate; UV/visible absorbance at 270 and 340 nm; and Brix, pH, EC, E_h_, and TN (in gN/L). Fig 2 was constructed using anodic voltammograms of CV for juice samples representing each of 24 cultivars (Section II of S1 File). Fig 2 only considers the potential range (0.7–1 V) of the primary CV peak in juice (Fig 1g). Fig 2 indicates strongly linear (red areas indicating positive correlation) relationships between anodic peak of juice samples at 0.9–1 V and following parameters: aromatic fluorescence fingerprint [28], sucrose and trans-aconitic acid concentrations, Brix, and UV/visible absorbance at 270 and 340 nm. Of those parameters, aromatic fluorescence fingerprint and UV/visible absorbance are directly related to polyphenols. Other parameters (sucrose, trans-aconitic acid, and Brix) are indirectly related to polyphenols, because concentrations of organic carbon products in juice are linearly correlated [28], as described in the subsequent paragraph dedicated to Fig 3. All other potential ranges and parameters evaluated in Fig 2 had negative r values or no significant linear correlations.

Fig 3 presents a Pearson’s correlation map of r values between all parameters investigated on juice and bagasse samples from the planting year 2017 in this and previous report [8]. Similarly to the planting year 2015 [28], parameters originating from the organic carbon products were positively correlated (red square labeled +r in Fig 3): concentrations of fructose, glucose, sucrose, and trans-aconitic acid; Brix, total organic carbon; UV/visible absorbance at 270 and 340 nm; contribution to aromatic fluorescence fingerprint; and E_pa_ by CV and CDPV. Those organic carbon parameters were negatively correlated (blue region marked–r in Fig 3) with tryptophan-like fluorescence fingerprint, electric conductivity, and area and height of small anodic (0.3–0.6 V) peak by CV and CDPV. In conclusion, E_pa_ is positively correlated with polyphenol, and negatively correlated with amino acid. This finding is in agreement with the contribution of polyphenols, rather than amino acid, on anodic peak of juice samples in Fig 2.

### Cultivar and planting month effects on the redox reactivity of polyphenols

Table 1 presents significant (p<0.05) cultivar (24 total in S1 Table in S1 File) and planting month effects and cultivar x planting month interactions on the electrochemical properties (CV and CDPV parameters and bulk E_h_). Both CV and CDPV employed 2 mm carbon SPE at 100 mV/s scan rate (an estimate for CDPV, as described in Materials and Methods). Planting month effects by post-hoc Tukey HSD (p<0.05) are presented as the increase (↑) or decrease (↓) from April to May (first arrow), and from May to June (second arrow) planting dates; one arrow indicate a significant difference only between April and June. Each variable is presented as the mean, standard deviation (s.d.), minimum, maximum, and number of non-zero values for n number of samples across three (April, May, and June) planting months for August-September harvest in 2017.

**Table 1:** Trapezoidal integration of major (0.5–1 V) and minor (0.3–0.6 V) anodic CV, CDPV peaks; E_h_ in mV; and UV/visible absorbance at wavelengths attributable to trans-aconitic acid and polyphenol [8]. Only significantly different p-value (<0.05) are given for the cultivar and planting month effects with post-hoc Tukey’s HSD test. Total number of samples (n for 24 cultivars × planting months (April, May, and June of 2017) × triplicate field plots), mean, standard deviation (s.d.), minimum, maximum, and number of non-zero values for each variable. Cultivar×planting interaction was observed only for the UV/visible absorbance at 270 nm (p = 0.02).

variable	n	mean	s.d.	min	max	non-zero	significant (<0.05) p value	
							cultivar	planting^a^
**Trapezoidal integrated areas of anodic derivative current in juice**								
CV (0.5-1V)	172	2.1E-06	7.5E-07	7.8E-07	5.5E-06	172	<0.00001 (No.5 Gambela>all others)*	<0.00001 (-↑)
CV (0.3–0.6V)^b^	163	6.5E-08	3.4E-08	1.4E-08	2.0E-07	163	0.00001 (Chinese>No.5 Gambela, Atlas, N109AxAtals, Isidomba, N111AxAtlas, N110AxAtlas;	<0.00001 (-↓)
							N110AxN98>No.5 Gambela, Atlas, Isidomba, N110AxAtlas; N98>Atlas; N111B>Atlas)	
CDPV (0.5-1V)	172	2.7E-06	8.4E-07	7.3E-07	7.2E-06	172	0.00002 (No.5 Gambela>N98, N111AxDale, N111AxIsidomba, Isidomba, N110AxDale, N111AxN98, Dale, N109AxIsidomba, N109AxChinese, N110AxAtlas;* Atlas>Isidomba, N111AxN98, Dale; Isidomba<N109AxAtlas, Chinese, N109B; Dale<N109AxAtlas, N109B)	<0.00001 (↓↑)
CDPV (0.3–0.6V)	171	2.1E-07	8.7E-08	3.5E-08	6.1E-07	171	0.00002 (N98>N110AxChinese, N111AxDale, Atlas, N109AxAtlas, Isidomba, N111AxN98, N109AxIsidomba, N110AxAtlas, N110AxIsidomba; N110AxN98>Isidomba, N111AxN98, N109AxIsidomba, N110AxAtlas; N111B>Isidomba)	<0.00001 (-↓)
**bulk reduction potential and UV/visible absorbance of juice**								
E_h_ (mV)	173	281	32	98	373	173		0.02 (↓-)
UV/vis 270nm (trans-aconitic acid)	174	0.95	0.48	0.20	2.44	174	<0.00001 (No.5 Gambela>all others;* Isidomba, Dale>Chinese, N111AxChinese, N111B; N109AxDale>Chinese, N111AxChinese, N111AxN98, N110B, N111B; Atlas>N111AxChinese)	<0.0001 (↑↑)
UV/vis 340nm (polyphenol)	174	0.18	0.09	0.05	0.67	174	<0.00001 (No.5 Gambela>all others)*	<0.00001 (↑-)

^a^Arrows indicate time trend (p<0.05 by post-hoc Tukey): increase (↑), decrease (↓), or no change (-) from April to May (first arrow), and from May to June (second arrow) planting dates; one arrow indicates significant difference only between April and June.

*Maximum in No.5 Gambela (p<0.05 by Tukey).

^b^If peak exists.

Trapezoidal integration was performed on CV and CDPV of each juice sample for both large (0.5–1 V) and small (0.3–0.6 V) anodic current peaks (S1 Fig in S1 File). Significant planting and cultivar (p<0.0001 for both) effects were observed for the large anodic peak (0.5–1 V) of CV. No.5 Gambela was significantly more electron-rich (larger integrated area of the primary anodic peak; marked * in Table 1) than all other cultivars. Later planting increased the electron donating capacity of juice (quantified as the anodic peak area), in agreement with the decreasing bulk E_h_ trend from April to May (Table 1). Opposite planting month effect (decrease from May to June) was observed for the small peak (0.3–0.6 V) area that was the highest for Chinese, compared to 6 other cultivars including No.5 Gambela. Trapezoidal integration of CDPV gave slightly different results from CV. For the large peak (0.5–1 V), No.5 Gambela (p<0.00001) was significantly more electron-rich than 9 other cultivars (as opposed to 23 others by CV). For the small peak (0.3–0.6 V), N98 had significantly (p = 0.0002) higher peak area than 9 other cultivars. Based on Fig 1, primary CV peak of juice could originate from tryptophan, tyrosine, or polyphenols. This peak was highest in No.5 Gambela, which contains the highest concentrations of trans-aconitic acid and aromatic structures, and has among the lowest concentrations of amino acids (as % contribution of tryptophan and tyrosine by EEM/PARAFAC) [8]. Because trans-aconitic acid is not electroactive with the method employed in the present study (Fig 1), the measured anodic current was determined to primarily originate from polyphenols. Using 2 mm SPE, CDPV enhanced the small peak (0.3–0.6 V) relative to CV (Section V of S1 File). The larger surface area (4x5 mm) of SPE further enhanced the minor peak (Section V of S1 File). Because only a small return (cathodic) peak was obtained for juice samples investigated in this study, CDPV was used for the Gaussian fitting of the cathodic peak (Section VI of S1 File).

In Table 2, Gaussian integration was performed on CV and CDPV to obtain electrochemical parameters: (1) peak area to quantify the amount of electron donated or accepted by each juice sample, (2) E_pa_ and E_pc_ to differentiate chemical structures and ring substituents responsible for oxidation/reduction (Fig 1 and S3 Table in S1 File), and (3) maximum peak current height in A (first derivative) at E_pa_ or E_pc_. For CV, Gaussian integration of anodic peak area (0.5–1 V) gave the same cultivar and planting month effects as trapezoidal integration (Tables 1 and 2): highest area of No.5 Gambela and an increase from May to June planting. Maximum peak height showed similar cultivar and planting month effects as the peak area, and No.5 Gambela was significantly higher than 8 other cultivars. Similarly to the anodic current area and height, No.5 Gambela had significantly higher (p<0.00001) E_pa_ (0.93±0.01 V as mean±standard error across planting months) than all other cultivars, except Dale (second highest E_pa_, 0.91±0.01 V) and Isidomba (0.90±0.01 V). Higher E_pa_ indicates higher aromaticity of polyphenolic structures, requiring more positive voltage for oxidation [31]. Although the cultivar x planting month interaction was significant (p = 0.04) for E_pa_, Tukey’s HSD did not differentiate the effects. In conclusion, No.5 Gambela had uniquely the highest anodic peak area and height, as well as E_pa_ (Table 2). Collectively, No.5 Gambela was the most electron-rich (peak area and height) and least easily oxidized (E_pa_) of all 24 cultivars investigated in S1 Table in S1 File. In addition, CV was more sensitive to differentiating cultivar and planting month effects than CDPV by both trapezoidal and Gaussian fits.

**Table 2:** Gaussian integration of CV anodic and CDPV anodic/cathodic peaks. Significant cultivar, planting month, and interaction p-values (<0.05 with post-hoc Tukey’s HSD test) for the fitted area, maximum height, and E_pa_ are provided.

variable	n	mean	s.d.	min	max	non-zero	significant (<0.05) p value		
							cultivar	planting^a^	interaction
**Gaussian integration of anodic and cathodic juice peaks**^c^									
CV anodic area (0.5-1V)	170	2.2E-06	7.6E-07	8.6E-07	4.9E-06	170	<0.00001 (No.5 Gambela>all others)*	<0.00001 (-↑)	
CV anodic max height (0.5-1V)	170	1.4E-05	3.9E-06	7.4E-06	2.6E-05	170	0.0001 (No.5 Gambela>all except 8 cultivars^d^^,^*; Chinese>Isidomba, N111AxN98, Dale)	<0.00001 (↓↑)	
CV anodic E_pa_ (0.5-1V)	170	0.88	0.03	0.82	0.95	170	0.00001 (No.5 Gambela>all except Isidomba, Dale;* Dale>all except No.5 Gambela,	<0.0001 (↑↑) N109AxDale, Atlas, N111AxIsidomba, Isidomba, N110AxAtlas; Isidomba>all except 9 cultivars;^e^ N109AxDale>N98)	0.04^f^
CDPV anodic area (0.5-1V)	171	2.7E-06	8.3E-07	3.3E-07	6.9E-06	171	0.0002 (No.5 Gambela>N98, N111AxIsidomba, N109AxN98, Isidomba, N110AxDale, N111AxN98, Dale;* Isidomba<Atlas, 109AxAtlas, N109B)	0.00006 (↓↑)	
CDPV anodic height (0.5-1V)	171	1.7E-05	5.1E-06	2.4E-06	4.4E-05	171	0.003 (Isidomba<No.5 Gambela, Atlas, 109AxAtlas, Chinese, 109B)	0.00001 (↓↑)	
CDPV anodic E_pa_ (0.5-1V)	171	0.80	0.04	0.43	0.86	171	0.04 (N109AxN98<No.5 Gambela, Isidomba)	0.00002 (↑-)	
CDPV anodic area (0.2–0.6V)^b^	160	2.2E-07	1.2E-07	0	8.1E-07	160	0.00004 (N98>all except N110AxN98, Chinese, N110AxDale, N111B, N109B)	0.01 (↓)	
CDPV anodic height (0.2–0.6V)^b^	160	1.7E-06	5.8E-07	2.4E-07	4.0E-06	160	0.00007 (N110AxN98>N111AxDale, Isidomba, N111AxN98, N109AxIsidomba, N110AxAtlas; N98>Isidomba, N111AxN98, N110AxAtlas)	<0.00001 (-↓)	0.04
CDPV anodic E_pa_ (0.2–0.6V)^b^	160	4.5E-01	3.5E-02	4.1E-01	7.9E-01	160		0.0007 (-↑)	
CDPV cathodic area^b^^,^^g^	96	1.0E-07	1.4E-07	0	8.2E-07	96		0.009 (↑↓)	
CDPV cathodic height^b^^,^^g^	96	9.2E-07	3.5E-07	9.2E-08	2.5E-06	96	0.00002 (No.5 Gambela>all others)*		
CDPV cathodic E_pa_^b^^,^^g^	96	0.75	0.03	0.57	0.79	96		0.0004 (-↓)	

^a^Arrows indicate time trend (p<0.05 by post-hoc Tukey): increase (↑), decrease (↓), or no change (-) from April to May (first arrow), and from May to June (second arrow) planting dates; one arrow indicates significant difference only between April and June.

^b^If peak exists.

^c^E_pa_ is the center of peak in volts; area is Gaussian-integrated current peak area at E_pa_; max height is the peak current height in amps at E_pa_.

^d^N110AxChinese, Atlas, N109AxAtlas, N111AxAtlas, Chinese, N111AxChinese, N109AxIsidomba, and N109B.

^e^No.5 Gambela, N111AxDale, N109AxDale, Atlas, N111AxIsidomba, N111AxAtlas, N110AxDale, Dale, N110AxAtlas, and N110AxIsidomba.

^f^No separation by Tukey.

^g^One-way ANOVA was employed (instead of factorial), because of low n.

For the large peak (0.5–1 V) of CDPV, No.5 Gambela had significantly (p = 0.0002) higher Gaussian-integrated area than 7 other cultivars including Dale and Isidomba. The E_pa_ values obtained by CDPV were lower than CV, and only cultivar effects were observed between the highest (0.84±0.01 V for No.5 Gambela and 0.82±0.01 V for Isidomba) and lowest (0.76±0.01 V for N109AxN98) cultivars. Gaussian integration of the small (0.2–0.6 V) peak showed similar cultivar effects as Trapezoidal integration (Table 1), where N98 had the highest peak area. For the peak height, significant interaction (p = 0.04) was observed, where May planting of N98 significantly differed from most of cultivars in June planting. No significant cultivar effects were observed for the E_pa_ of small (0.2–0.6 V) anodic peak by CDPV.

Because only 96 of 171 CDPV voltammograms had cathodic return peaks (0.5–1 V), one-way ANOVA (with cultivar and planting month as the categorical factors, instead of factorial ANOVA) was carried out for the cathodic parameters of CDPV in Table 2. Cultivar effects were only observed for the peak height of cathodic return CDPV. No.5 Gambela had significantly higher cathodic current height (p = 0.00002) than all other cultivars, similarly to the Gaussian and trapezoidal integration of corresponding anodic current area by CV at 0.5–1 V. The return peak is attributable to the reduction of oxidized polyphenols in No.5 Gambela.

In Table 1, UV/visible absorbance of juice was analyzed at selected wavelengths to determine the cultivar effects of polyphenols at 340 nm, where trans-aconitic acid does not absorb. At 340 nm where only polyphenols absorb significantly, No.5 Gambela had significantly higher absorbance (0.37±0.02, p<0.00001) than all other cultivars. This trend follows that of primary anodic CV areas used to measure polyphenol electrochemistry. At lower wavelength (270 nm) where trans-aconitic acid absorbs significantly, cultivar and planting month effects were similar to 340 nm. Therefore, trans-aconitic acid and polyphenols likely have similar cultivar and planting month effects. At 270 nm, significant cultivar x planting month interaction was observed, especially between April and May plantings (p = 0.02).

**Fig 4:**
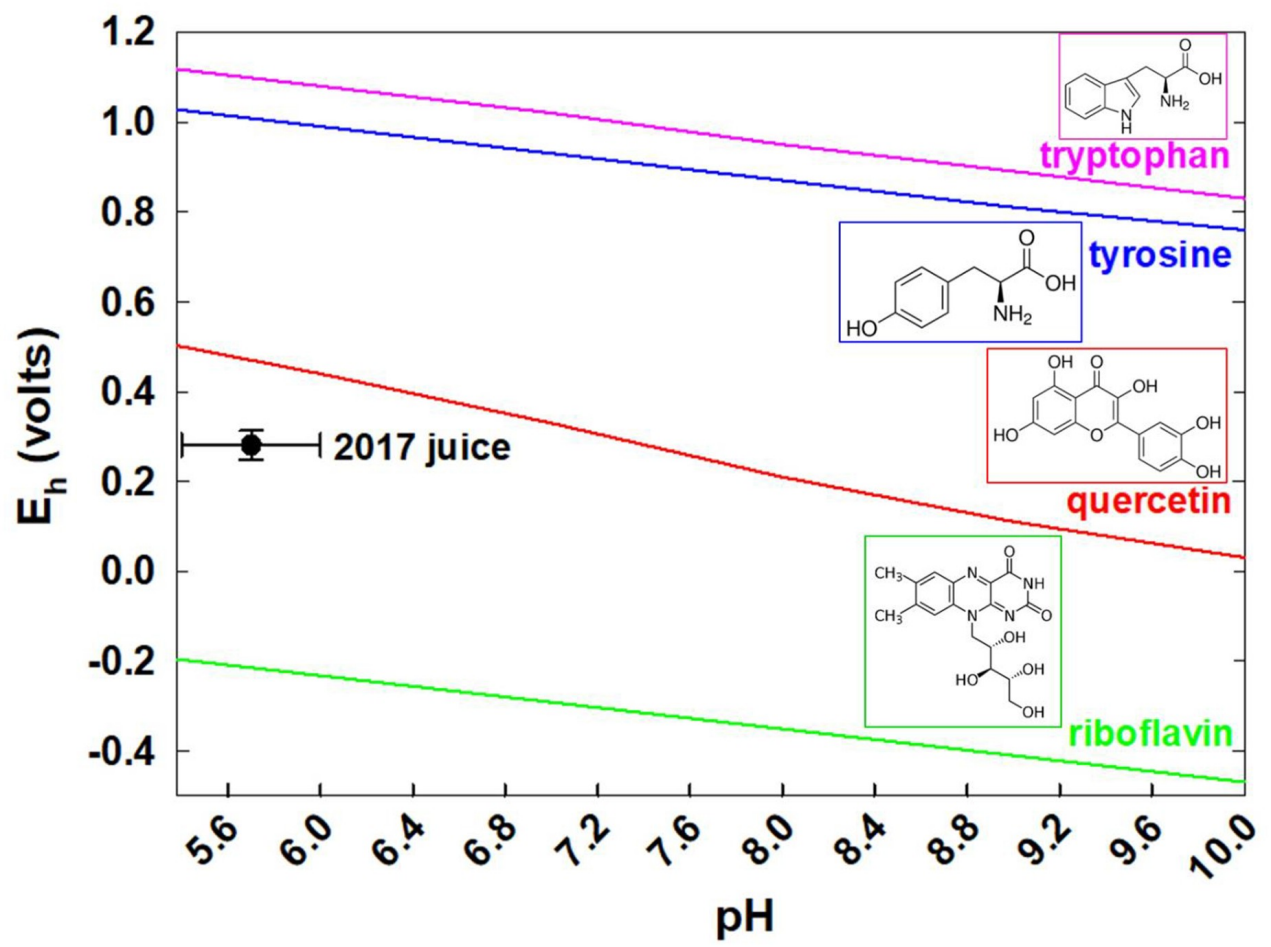
E_h_-pH diagram for one-electron oxidation/reduction of tryptophan (pink line), tyrosine (blue line) [32], quercetin (red line) [33], riboflavin (green line) [34], and sweet sorghum juice samples from 2017 planting year (black symbol) against the standard hydrogen electrode. Error bars for the 2017 juice represents mean±s.d. for 173 samples in Table 2. Literature values were used for amino acids (in 0.2 M KCl, pH 13) [32], quercetin (in 0.5 M KCl; ethylene glycol as co-solute) [31, 33, 39], and riboflavin (in 0.1 M NaHCO_2_) [34]. Literature E_h_-pH values were reported against NHE. Experimental E_h_ value of juice samples could be converted to SHE scale by adding 200±60mV.

Fig 4 presents an E_h_-pH diagram for one-electron oxidation of tryptophan (to form indole radical) [31], tyrosine (phenoxyl radical) [32], quercetin (radical of catechol in B ring) [33], and riboflavin (semiquinone) [34], all reported in the literature against normal hydrogen electrode (NHE). Reduction potentials of quercetin and riboflavin in Fig 4 was obtained kinetically (radical generation and spectrophotometric quantification) by pulse radiolysis at fixed pH [33], while CV was employed for tryptophan and tyrosine. Standard hydrogen electrode (SHE) is an ideal (theoretical) electrode with H^+^ at unity activity saturated with H_2_ gas at unity fugacity [35, 36]. NHE is a different reference electrode containing 1 atm partial pressure H_2_ and 1M H_2_SO_4_ [37]. Degree of deviation from the standard state is unknown in experiments employing NHE, where activity of different ionic solution components cannot be controlled [35–37].

Those literature values are compared against E_h_ and pH values of sweet sorghum juice samples from 2017 planting year (black symbol as mean±s.d. for 173 samples in Table 1). Experimental E_h_ value of juice samples could be converted to SHE scale by adding 200±60mV, as described in Materials and Methods. At pH 5.7±0.3, the E_h_ value of sweet sorghum (0.281±0.032 V) is lower than amino acids, higher than riboflavin, and closest to quercetin. The, E_h_, pH, and EC of 2017 juice samples all decreased with the planting month (Table 1) [8]. As shown in Table 1, there was no statistically significant (p<0.05) difference in E_h_ (and pH provided in our previous report [8]) values among different cultivars.

Quercetin has the lowest one-electron reduction potential among flavonoids (e.g., catechin, galangin), because the electron-donating hydroxyl group in the O-3 position of the C ring is conjugated with the catechol B radical through the 2,3 double bond [33]. Observation of E_h_-pH behavior in Fig 4 suggests that semiquinone radical [3, 4] of quercetin-like polyphenolic structure is responsible for the redox reactivity of sweet sorghum. The E_h_-pH trend of plant phenolics is traditionally investigated by manually changing the pH of a given sample by adding acid or base [38]. Instead, Fig 4 (filled circle labeled “2017 juice”) presents intrinsic E_h_ and pH values of 173 sweet sorghum juice samples collected from the field experiments, without modifying pH. Although thermodynamic driving force does not predict the kinetic lability, polyphenols in sweet sorghum (Fig 4) are capable of reducing the primary amino acids in juice, and other chemical structures having higher reduction potentials.

## Supporting information

S1 File Sections I-VI: Methods; representative juice samples (24 total matching S1 Table) used to calculate Pearson’s correlation coefficient across 0.7–1 V range of CV anodic voltammogram; Gaussian fitting parameters for the authentic standards; representative cultivars enriched with amino acids and polyphenols; influence of SPE area and methods (CV, CDPV) on peak properties; representative anodic and cathodic CDPV peaks subjected to integration. (DOC)

## References

[1] USCP. Food-Grade Sorghum Has Changed to Meet Growing Consumer Demand. [Online Article] https://www.sorghumcheckoff.com/news-and-media/newsroom/2019/01/16/food-grade-sorghum-has-changed-to-meet-growing-consumer-demand/ (accessed 12 April 2019). United Sorghum Checkoff Program (USCP), Lubbock, TX: 2019 Contract No.: 13 March 2018.

[2] Manach C, Scalbert A, Morand C, Rémésy C, Jiménez L. Polyphenols: Food sources and bioavailability. Am J Clin Nutr. 2004;79(5):727–47. 10.1093/ajcn/79.5.727 15113710

[3] Uchimiya M, Stone AT. Redox reactions between iron and quinones: Thermodynamic constraints. Geochimica et Cosmochimica Acta. 2006;70(6):1388–401. 10.1016/j.gca.2005.11.020

[4] Uchimiya M, Stone AT. Reversible redox chemistry of quinones: Impact on biogeochemical cycles. Chemosphere. 2009;77(4):451–8. 10.1016/j.chemosphere.2009.07.025 19665164

[5] Tan SLJ, Webster RD. Electrochemically induced chemically reversible proton-coupled electron transfer reactions of riboflavin (Vitamin B_2_). J Am Chem Soc. 2012;134(13):5954–64. 10.1021/ja300191u 22390470

[6] Gupta A, Srivastava S. Genotypic differences in nutritional quality of sprouted sorghum (Sorghum bicolor) flour. J Food Sci Technol. 1997;34(1):56–8.

[7] Uchimiya M, Knoll JE, Harris-Shultz KR. Electrochemical evaluation of sweet sorghum fermentable sugar bioenergy feedstock. ACS Sustainable Chem Eng. 2017;5(8):7352–64. 10.1021/acssuschemeng.7b01662

[8] Uchimiya M, Knoll JE. Accumulation of carboxylate and aromatic fluorophores by a pest-resistant sweet sorghum [*Sorghum bicolor (L*.*) Moench*] genotype. ACS Omega. 2019;4(24):20519–29. 10.1021/acsomega.9b02267 31858036 PMC6906763

[9] Guden B, Yol E, Ikten C, Erdurmus C, Uzun B. Molecular and morphological evidence for resistance to sugarcane aphid (Melanaphis sacchari) in sweet sorghum [Sorghum bicolor (L.) Moench]. 3 Biotech. 2019;9(6). 10.1007/s13205-019-1783-8 31168438 PMC6546771

[10] Dreyer DL, Reese JC, Jones KC. Aphid feeding deterrents in sorghum—Bioassay isolation and characterization. J Chem Ecol. 1981;7(2):273–84. 10.1007/BF00995750 24420473

[11] Gulaboski R, Spirevska I, Šoptrajanova L, Slavevska R. Square-wave voltammetric method for determination of fumaric and maleic acid—Determination of fumaric acid in wine. Anal Lett. 2001;34(10):1719–31. 10.1081/AL-100105355

[12] El-Cheikh FM, Rashwan FA, Mahmoud HA, El-Rouby M. Electrochemical response of the two isomers conjugated acids, maleic and fumaric, on glassy carbon electrode modified with platinum nanoparticles. J Appl Electrochem. 2010;40(1):79–89. 10.1007/s10800-009-9983-2

[13] Spirevska I, Šoptrajanova L, Gulaboski R. Square-wave voltammetric method for determination of aconitic acid. Anal Lett. 2000;33(5):919–28.

[14] Escobar JD, Alcaniz M, Masot R, Fuentes A, Bataller R, Soto J, et al Quantification of organic acids using voltammetric tongues. Food Chem. 2013;138(2–3):814–20. 10.1016/j.foodchem.2012.11.078 23411182

[15] Nayeri S, Alizadeh T. Evaluation of graphite/AgCl composite as a new and highly efficient electrocatalyst for electroxidation of oxalic acid. Anal Bioanal Electrochem. 2018;10(7):840–50.

[16] Yoo JS, Park SM. Programmed potential sweep voltammetry for lower detection limits. Anal Chem. 2005;77(11):3694–9. 10.1021/ac0481598 15924407

[17] Hoyos-Arbeláez J, Vázquez M, Contreras-Calderón J. Electrochemical methods as a tool for determining the antioxidant capacity of food and beverages: A review. Food Chem. 2017;221:1371–81. 10.1016/j.foodchem.2016.11.017 27979102

[18] Berggren M, Malliaras GG. How conducting polymer electrodes operate. Science. 2019;364(6437):233–4. 10.1126/science.aaw9295 31000650

[19] Bard AJ, Faulkner LR. Electrochemical methods: Fundamentals and Applications. Second ed New York, NY: John Wiley & Sons; 2001.

[20] Hermans A, Keithley RB, Kita JM, Sombers LA, Wightman RM. Dopamine detection with fast-scan cyclic voltammetry used with analog background subtraction. Anal Chem. 2008;80(11):4040–8. 10.1021/ac800108j 18433146

[21] Uchimiya M, Knoll JE, Anderson WF, Harris-Shultz KR. Chemical analysis of fermentable sugars and secondary products in 23 sweet sorghum cultivars. Journal of Agricultural and Food Chemistry. 2017;65(35):7629–37. 10.1021/acs.jafc.7b00675 28771348

[22] Uchimiya M, Knoll JE. Prediction of carboxylic and polyphenolic chemical feedstock quantities in sweet sorghum. Energ Fuel. 2018;32(4):5252–63. 10.1021/acs.energyfuels.8b00491

[23] USDA, ARS, National Genetic Resources Program. Germplasm Resources Information Network—(GRIN). [Online Database] National Germplasm Resources Laboratory, Beltsville, Maryland. http://www.ars-grin.gov/cgi-bin/npgs/acc/display.pl?1201506 (accessed 29 August 2018) USDA, ARS, National Genetic Resources Program.

[24] Wu X, Staggenborg S, Propheter JL, Rooney WL, Yu J, Wang D. Features of sweet sorghum juice and their performance in ethanol fermentation. Ind Crop Prod. 2010;31(1):164–70. 10.1016/j.indcrop.2009.10.006

[25] Abraham MH, Acree WE Jr. On the solubility of quercetin. Journal of Molecular Liquids. 2014;197:157–9. 10.1016/j.molliq.2014.05.006

[26] Elgrishi N, Rountree KJ, McCarthy BD, Rountree ES, Eisenhart TT, Dempsey JL. A practical beginner’s guide to cyclic voltammetry. J Chem Educ. 2018;95(2):197–206. 10.1021/acs.jchemed.7b00361

[27] Vilas-Boas Â, Valderrama P, Fontes N, Geraldo D, Bento F. Evaluation of total polyphenol content of wines by means of voltammetric techniques: Cyclic voltammetry vs differential pulse voltammetry. Food Chem. 2019;276:719–25. 10.1016/j.foodchem.2018.10.078 30409654

[28] Uchimiya M, Knoll JE. Rapid data analytics to relate sugarcane aphid [(*Melanaphis sacchari* (Zehntner)] population and damage on sorghum (*Sorghum bicolor* (L.) Moench). Sci Rep. 2019;9(1). 10.1038/s41598-018-36815-0 30674945 PMC6344576

[29] Hendrickson HP, Kaufman AD, Lunte CE. Electrochemistry of catechol-containing flavonoids. J Pharm Biomed Anal. 1994;12(3):325–34. 10.1016/0731-7085(94)90007-8 8031931

[30] Brett AMO, Ghica ME. Electrochemical oxidation of quercetin. Electroanalysis. 2003;15(22):1745–50. 10.1002/elan.200302800

[31] Wardman P. Reduction potentials of one electron couples involving free radicals in aqueous solution. J Phys Chem Ref Data. 1989;18(4):1637–755. 10.1063/1.555843

[32] Harriman A. Further comments on the redox potentials of tryptophan and tyrosine. J Phys Chem. 1987;91(24):6102–4. 10.1021/j100308a011

[33] Jovanovic SV, Steenken S, Hara Y, Simic MG. Reduction potentials of flavonoid and model phenoxyl radicals. Which ring in flavonoids is responsible for antioxidant activity? J Chem Soc, Perkin Trans 2. 1996;(11):2497–504. 10.1039/p29960002497

[34] Meisel D, Neta P. One-electron reduction potential of riboflavine studied by pulse radiolysis. J Phys Chem. 1975;79(23):2459–61. 10.1021/j100590a002

[35] Duran-Chaves M, Sanabria-Chinchilla J. From Ideality to Simplicity: A Robust and Affordable Hydrogen Reference Electrode. J Chem Educ. 2020 10.1021/acs.jchemed.9b00664

[36] Biegler T, Woods R. The standard hydrogen electrode a misrepresented concept. J Chem Educ. 1973;50(9):604–5.

[37] Ramette RW. Textbook forum: Outmoded terminology: The normal hydrogen electrode. J Chem Educ. 1987;64(10):885.

[38] Hagerman AE, Riedl KM, Jones GA, Sovik KN, Ritchard NT, Hartzfeld PW, et al High molecular weight plant polyphenolics (tannins) as biological antioxidants. Journal of Agricultural and Food Chemistry. 1998;46(5):1887–92. 10.1021/jf970975b 29072454

[39] Steenken S, Neta P. One-electron redox potentials of phenols. Hydroxy- and aminophenols and related compounds of biological interest. J Phys Chem. 1982;86(18):3661–7. 10.1021/j100215a033

