# Supplementary material for: Electroactivity of polyphenols in sweet sorghum (Sorghum bicolor (L.) Moench) cultivars
Source: PLoS One. 2020 Jul 14;15(7):e0234509. doi: 10.1371/journal.pone.0234509 (PMC7360041; doi:10.1371/journal.pone.0234509)
Supplement: S1 File — (DOC) [file pone.0234509.s001.doc]

**I. Methods**

**Field experiment.** In 2017, a field experiment was conducted near Tifton, GA (31° 29’ N, 83° 31’ W) on a Tifton loamy sand (fine-loamy, kaolinitic, thermic Plinthic Kandiudults) with the following modifications to the experimental design described in detail previously (Knoll & Anderson, 2016; M. Uchimiya, J. E. Knoll, & K. R. Harris-Shultz, 2017a). Fifteen hybrids were generated by paper bag pollination using male-sterile female seed parents (N109A, N110A, and N111A) (Gorz, Haskins, & Johnson, 1990), each crossed to five inbred lines (Atlas, Chinese, Dale (Broadhead & Coleman, 1973), Isidomba, and N98) (Gorz et al., 1990) as males. The inbred parents, including male-fertile (B-line) versions of the female lines, were also planted. A sugarcane aphid resistant control No.5 Gambela (PI 257599) was also included, for a total of 24 entries (Table S1) planted in a split-plot design with three replications with planting dates (April, May, or June) as the main plot factor and cultivars as the subplot factor. Planting dates were April 24, May 17, and June 30, 2017. Each subplot consisted of a single row, 6 m long with 0.9 m between rows. Subplots consisted of two rows in 2015, but were reduced to one row in 2016 and 2017 due to a limited supply of seed. Prior to planting, granular N-P-K (10-10-10) fertilizer was applied at a rate of 560 kg ha^-1^. At ≈30 d after planting, additional N was applied by a side-dress liquid application (112 kg ha^-1^ N). Weeds were controlled by the herbicides pendimethalin (N-(1-ethylpropyl)-3,4-dimethyl-2,6-dinitrobenzenamine), atrazine (1-chloro-3-ethylamino-5-isopropylamino-2,4,6-triazine), and bentazon (3-isopropyl-1H-2,1,3-benzothiadiazin-4(3H)-one 2,2-dioxide). Irrigation was applied only as needed to speed germination, and no insecticides were used.

Harvest dates were set to the hard-dough stage of maturity (when Brix typically peaks), and occurred on July 25, August 1, 8, 15, 23, 29, September 5, 14, October 3, 10, 17, 25, and November 1 in 2017. Three representative stalks were harvested from each subplot, panicles and leaves were removed, and juice was extracted from the stems by passing twice through a portable three-roller mill (Sor-Cane Porta-Press, McClune, Reynolds, GA). Juice samples were immediately frozen after measuring the soluble solids concentration (Brix) using a digital refractometer (Refracto 30GS, Mettler-Toledo, Columbus, OH) (Knoll et al., 2016). The bagasse portion was dried at 60 °C until the weight stabilized, ground in a Wiley mill (Thomas Scientific, Swedesboro, NJ), and sieved (<2 mm).

**Table S1.** Inbred and hybrid sweet sorghum varieties investigated in this study. Entries are color-coded to correspond to the figures. PI number was obtained from the National Plant Germplasm System (NPGS) accession identifier (https://www.ars-grin.gov). Table was modified from (M. Uchimiya, J. E. Knoll, & K. R. Harris-Shultz, 2017b).

| Entry | Group membership | PI number | Reference |
| --- | --- | --- | --- |
| Atlas | Inbred male, sweet/forage cultivar | PI 641807 | (Knoll et al., 2016) |
| Chinese | Inbred male, landrace | - | (Knoll et al., 2016) |
| Dale | Inbred male, improved sweet cultivar | PI 651495 | (Broadhead et al., 1973) |
| Isidomba | Inbred male, landrace | PI 144331 | (Knoll et al., 2016) |
| N98 | Inbred male, improved breeding line | PI 535783 | (Gorz et al., 1990) |
| N109B | Inbred female, 3-dwarf seed parent | PI 535794 | (Gorz et al., 1990) |
| N110B | Inbred female, 2-dwarf seed parent | PI 535795 | (Gorz et al., 1990) |
| N111B | Inbred female, 2-dwarf seed parent | PI 535796 | (Gorz et al., 1990) |
| N109A x Atlas | Hybrid | - | (Knoll et al., 2016) |
| N109A x Chinese | Hybrid | - | (Knoll et al., 2016) |
| N109A x Dale | Hybrid | - | - |
| N109A x Isidomba | Hybrid | - | (Knoll et al., 2016) |
| N109A x N98 | Hybrid | - | (Knoll et al., 2016) |
| N110A x Atlas | Hybrid | - | (Knoll et al., 2016) |
| N110A x Chinese | Hybrid | - | (Knoll et al., 2016) |
| N110A x Dale | Hybrid | - | - |
| N110A x Isidomba | Hybrid | - | (Knoll et al., 2016) |
| N110A x N98 | Hybrid | - | (Knoll et al., 2016) |
| N111A x Atlas | Hybrid | - | (Knoll et al., 2016) |
| N111A x Chinese | Hybrid | - | (Knoll et al., 2016) |
| N111A x Dale | Hybrid | - | - |
| N111A x Isidomba | Hybrid | - | (Knoll et al., 2016) |
| N111A x N98 | Hybrid | - | (Knoll et al., 2016) |
| No.5 Gambela | Landrace zerazera | PI 257599 | (Rosenow et al., 1997; Wang et al., 2009) |

**II. Representative juice samples (24 total matching Table S1) used to calculate Pearson’s correlation coefficient across 0.7-1 V range of CV anodic voltammogram**

**Table S2.** Samples for Figure 2

| **plot** | **pedigree** | **planting** |
| --- | --- | --- |
| 101 | No. 5 Gambela | 3 |
| 102 | N98 | 3 |
| 136 | N110A x Chinese | 3 |
| 137 | Dale | 3 |
| 143 | N 111 B | 1 |
| 144 | N110A x Atlas | 1 |
| 147 | N 109 B | 3 |
| 157 | N111A x N98 | 2 |
| 159 | Atlas | 3 |
| 205 | N109A x Chinese | 1 |
| 219 | N111A x Atlas | 1 |
| 242 | N 110 B | 1 |
| 255 | N109A x N98 | 1 |
| 265 | N109A x Atlas | 1 |
| 266 | N109A x Isidomba | 1 |
| 271 | N111A x Chinese | 3 |
| 318 | Isidomba | 3 |
| 112 | N111A x Isidomba | 3 |
| 332 | N109A x Dale | 3 |
| 340 | N110A x N98 | 1 |
| 342 | Chinese | 3 |
| 357 | N110A x Isidomba | 1 |
| 366 | N111A x Dale | 3 |
| 367 | N110A x Dale | 3 |

**III. Gaussian fitting parameters for the authentic standards**

**Table S3.** Comparison of anodic CV and CDPV peak position and area for authentic standards and juice (n=170 from Table 2). Data for quercetin and catechin are literature values; only CV analysis was conducted for those compounds.(Uchimiya et al., 2017a)

| **compound** | **Gaussian CV** | | **Gaussian CDPV** | |
| --- | --- | --- | --- | --- |
|  | **E_pa_ (V)** | **area** | **E_pa_ (V)** | **area** |
| quercetin anodic | 0.11 | 1.25E-06 |  |  |
| 0.5mM in ethanol | 0.26 | 7.93E-07 |  |  |
|  | 0.59 | 1.57E-06 |  |  |
|  | 0.99 | 5.43E-06 |  |  |
|  | **sum** | **9.04E-06** |  |  |
| catechin anodic | -0.48 | 1.61E-06 |  |  |
| 0.5mM in ethanol | 0.30 | 3.12E-06 |  |  |
|  | 0.69 | 2.75E-06 |  |  |
|  | **sum** | **7.48E-06** |  |  |
| tryptophan anodic (1mM in DDW) | 0.83 | 8.53E-06 | 0.73 | 1.08E-05 |
| tyrosine anodic (1mM in DDW) | 0.77 | 5.17E-06 | 0.71 | 4.24E-06 |
| juice anodic (mean±s.d.) | 0.88±0.03 | 2.2E-6±7.6E-7 | 0.80±0.04 | 2.7E-6±8.3E-7 |

**IV. Representative cultivars enriched with amino acids and polyphenols**


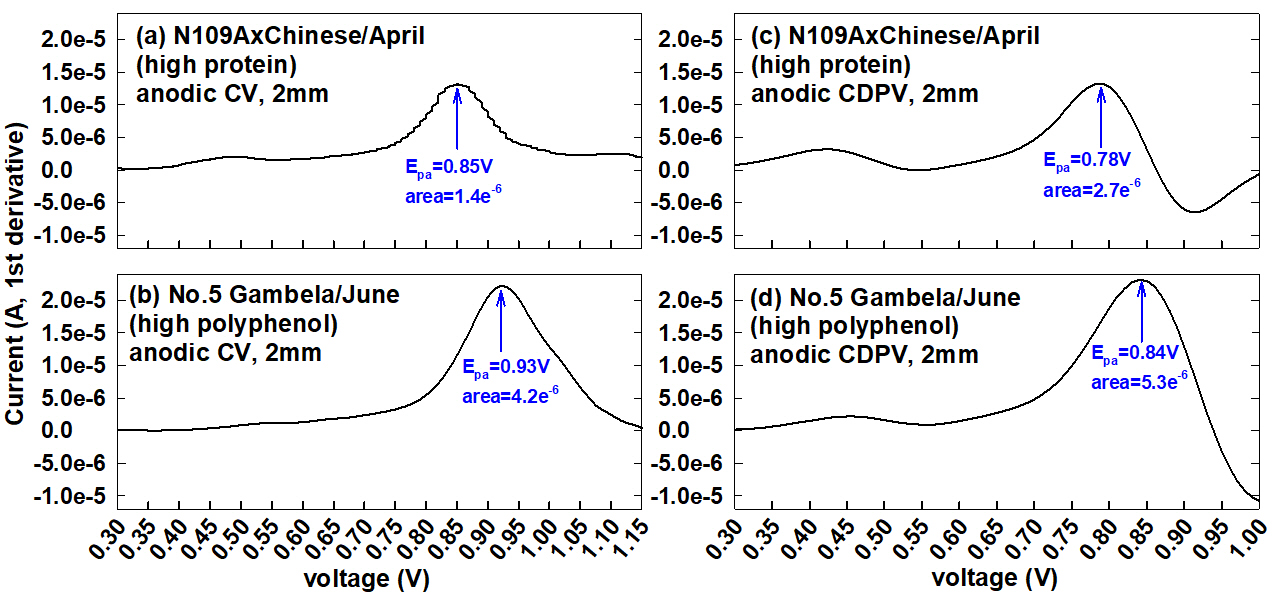


**Figure S1.** Anodic CV (a, b) and CDPV (c, d; both after background subtraction and first derivative with smoothing) of representative juice samples with high tryptophan/tyrosine (N109AxChinese/April in a and c) or polyphenol (No.5 Gambela/June in c and d) contents. Higher E_pa_ and peak area are observed in No.5 Gambela/June (bottom) than N109AxChinese/April (top) for both CV (left) and CDPV (right).

**V. Influence of SPE area and methods (CV, CDPV) on peak properties**

Anodic CV (2 mm SPE) and CDPV (2 and 4x5 mm) of N110AxIsidomba (June):

**VI. Representative anodic and cathodic CDPV peaks subjected to integration**

Cathodic CDPV of No.5 Gambela (April). Shaded area was integrate by Gaussian fitting:

Anodic CDPV of No.5 Gambela (April). Shaded area is the “small” peak for trapezoidal/Gaussian integration:

Anodic CDPV of No.5 Gambela (April). Shaded area is the “large” peak for trapezoidal/Gaussian integration:

**References**
